# Supplementary material for: The Burden of Hospitalization and Rehospitalization Among Patients Hospitalized with Severe Community-Acquired Bacterial Pneumonia in the United States, 2018–2022
Source: Antibiotics (Basel). 2025 Jun 25;14(7):642. doi: 10.3390/antibiotics14070642 (PMC12291879; doi:10.3390/antibiotics14070642)
Supplement: Supplementary file 1 [file antibiotics-14-00642-s001.zip › antibiotics-3620996-supplementary.pdf]

Supplemental material

File S1: ICD-10 codes used to identify CABP

A01.03	Typhoid pneumonia
A02.22	Salmonella pneumonia
A21.2	Pulmonary tularemia
A22.1	Pulmonary anthrax
A31.0	Pulmonary mycobacterial infection
A37.01	Whooping cough due to *Bordetella pertussis* with pneumonia
A37.11	Whooping cough due to *Bordetella parapertussis* with pneumonia
A37.81	Whooping cough due to other *Bordetella* species with pneumonia
A37.91	Whooping cough, unspecified species with pneumonia
A42.0	Pulmonary actinomycosis
A43.0	Pulmonary nocardiosis
A48.1	Legionnaires' disease
A50.04	Early congenital syphilitic pneumonia
A54.84	Gonococcal pneumonia
A70	*Chlamydia psittaci* infections
J13	Pneumonia due to *Streptococcus pneumoniae*
J14	Pneumonia due to *Hemophilus influenzae*
J15	Bacterial pneumonia, not elsewhere classified
J15.0	Pneumonia due to *Klebsiella pneumoniae*
J15.1	Pneumonia due to *Pseudomonas*
J15.2	Pneumonia due to *Staphylococcus*

J15.20	Pneumonia due to *Staphylococcus*, unspecified
J15.21	Pneumonia due to *Staphylococcus aureus*
J15.211	Pneumonia due to methicillin-susceptible *Staphylococcus aureus*
J15.212	Pneumonia due to methicillin-resistant *Staphylococcus aureus*
J15.29	Pneumonia due to other *Staphylococcus*
J15.3	Pneumonia due to *Streptococcus*, group b
J15.4	Pneumonia due to other *Streptococci*
J15.5	Pneumonia due to *Escherichia coli*
J15.6	Pneumonia due to other gram-negative bacteria
J15.7	Pneumonia due to *Mycoplasma pneumoniae*
J15.8	Pneumonia due to other specified bacteria
J15.9	Unspecified bacterial pneumonia
J16.0	Chlamydial pneumonia
P23.1	Congenital pneumonia due to *Chlamydia*
P23.2	Congenital pneumonia due to *Staphylococcus*
P23.3	Congenital pneumonia due to *Streptococcus*, group b
P23.4	Congenital pneumonia due to *Escherichia* coli
P23.5	Congenital pneumonia due to *Pseudomonas*
P23.6	Congenital pneumonia due to other bacterial agents

File S2. Patient selection schema

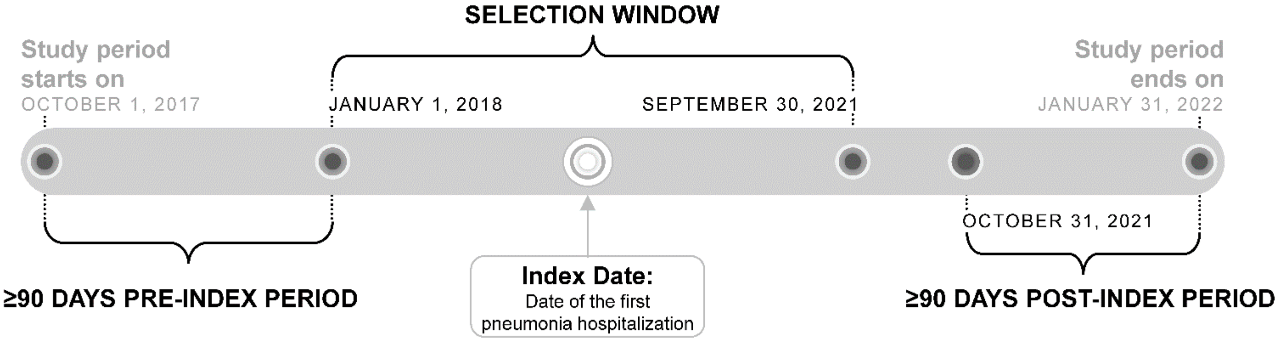

File S3: Seasonal variation in sCABP admissions

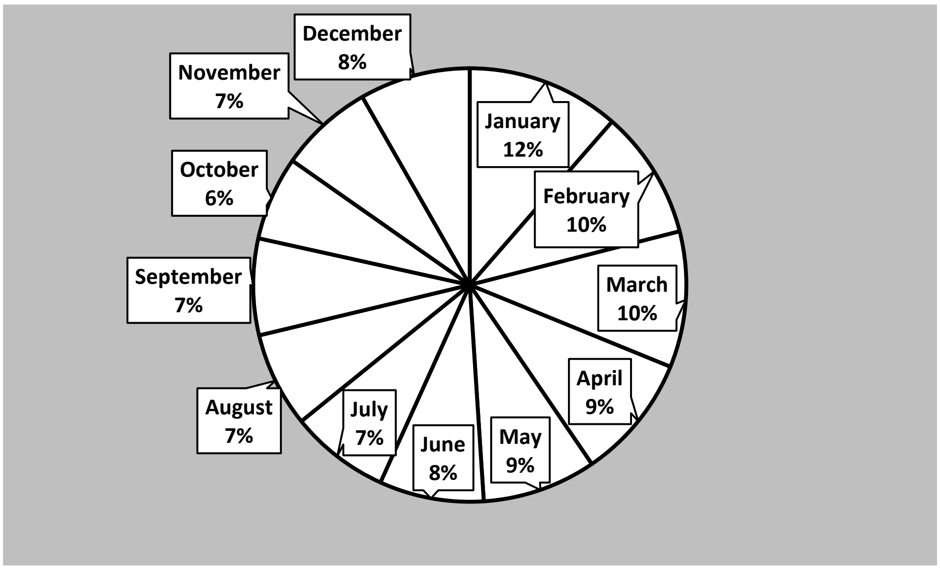

File S4: Individual comorbidities

	(N=24,422)	
	n	%
**Comorbid condition**		
Alcohol/drug abuse	932	3.82 %
Asthma	684	2.80 %
Cardiac arrhythmia	3,194	13.08 %
Cardiac valvular disease	827	3.39 %
Cerebrovascular disease	1,663	6.81 %
Chronic kidney disease	2,659	10.89 %
Chronic pain/fibromyalgia	621	2.54 %
Congestive heart failure	2,793	11.44 %
COPD	3,151	12.90 %
COVID-19	202	0.83 %
Dementia/Alzheimer's disease	918	3.76 %
Depression	1,421	5.82 %
Diabetes mellitus	3,831	15.69 %
Dyslipidemia	3,713	15.20 %
Epilepsy/seizure disorder	697	2.85 %
Hepatitis	531	2.17 %
HIV/AIDS	126	0.52 %
Hypertension	4,074	16.68 %
Liver/gallbladder/pancreatic disease	1,100	4.50 %
Myocardial infarction/coronary artery disease	2,958	12.11 %

Osteoarthritis	1,237	5.07 %
Paralysis	368	1.51 %
Peptic ulcer	173	0.71 %
Peripheral vascular disease	586	2.40 %
Pregnancy	9	0.04 %
Renal failure/dialysis	2,579	10.56 %
Rheumatic disease	318	1.30 %
Schizophrenia	230	0.94 %
Severe hepatic impairment	0	0.00 %
Sleep disorders	1,292	5.29 %
Smoking	4,474	18.32 %
Thyroid disease	1,532	6.27 %

AIDS, acquired immune deficiency syndrome; COPD, chronic obstructive pulmonary disease; HIV, human immunodeficiency virus.

File S5. Medications received within 90 days prior to index hospitalization

	(N=24,422)	
	**n**	**%**
Immunosuppressive drugs	138	0.57 %
Chemotherapy	679	2.78 %
Inhalers for lung disease	2,665	10.91 %
Antibiotics		
Aminoglycosides	143	0.59 %
Antifungals	354	1.45 %
Beta-lactams	1,435	5.88 %
Carbapenems	445	1.82 %
Cephalosporins	2,996	12.27 %
Fluoroquinolones	879	3.60 %
Folate pathway inhibitors	3	0.01 %
Glycopeptides	1,561	6.39 %
Macrolides	923	3.78 %
Monobactams	70	0.29 %
Monoclonal antibodies	6	0.02 %
Other antibiotics	585	2.40 %
Oxazolidinones	121	0.50 %
Penicillin	127	0.52 %
Penicillin with beta-lactamase inhibitors	1,368	5.60 %
Polymyxins	42	0.17 %

Tetracyclines	342	1.40 %

File S6. Top 5 diagnoses and proportions of CABP diagnoses during 30- and 90-day readmission periods

	(N=24,422)	
	**n**	**%**
**30-day readmissions**		
R06: Abnormalities of breathing	463	11.34 %
A41: Other sepsis	415	10.17 %
J96: Respiratory failure, not elsewhere classified	383	9.38 %
Z51: Encounter for other aftercare and medical care	217	5.32 %
J18: Pneumonia, unspecified organism	172	4.21 %
**90-day readmissions**		
R06: Abnormalities of breathing	718	11.68 %
A41: Other sepsis	643	10.46 %
J96: Respiratory failure, not elsewhere classified	502	8.17 %
J18: Pneumonia, unspecified organism	264	4.29 %
R41: Other symptoms and signs involving cognitive functions and awareness	240	3.90 %
